# PReS-FINAL-2331: Low-penetrance NLRP3 variants

**DOI:** 10.1186/1546-0096-11-S2-P321

**Published:** 2013-12-05

**Authors:** T Endres, F Hofer, R Goldbach-Mansky, HM Hoffman, N Blank, K Krause, C Rietschel, G Horneff, P Lohse, J Kuemmerle-Deschner

**Affiliations:** 1 Department of Pediatrics, Division of Pediatric Rheumatology, University Hospital Tuebingen, Tuebingen, Germany; 2 Translational Autoinflammatory Disease Section, NIAMS/NIH, Bethesda, MD, USA; 3 Division of Rheumatology and Allergy/Immunology, University of California at San Diego, San Diego, CA, USA; 4 Hämatologie, Onkologie u. Rheumatologie, Universitätsklinikum Heidelberg, Heidelberg, Germany; 5 "Allergie-Centrum Charité", Klinik für Dermatologie, Charité Campus Mitte, Berlin, Germany; 6 Kinder-und Jugendrheumatologie, Clementine-Kinderhospital, Frankfurt, Germany; 7 Abteilung für Allgemeine Kinder-und Jugendmedizin, Asklepios-Klinik Sankt Augustin, Sankt Augustin, Germany; 8 Institut für Laboratoriumsmedizin und Humangenetik, Singen, Germany

## Introduction

Cryopyrin-associated periodic syndrome (CAPS) presents as rare, autosomal dominant disease spectrum, due to mutations in the *NLRP3*-gene which lead to excessive interleukin-1 (IL-1) release.

In patients with low-penetrance NLRP3 variants, the clinical presentation varies widely. So far, a correlation with a specific phenotype could not be demonstrated.

## Objectives

The aim of this study was to analyze the association of the V198M, R488K, and Q703K substitutions with a specific phenotype, laboratory markers, and the response to IL-1 inhibitors anakinra, canakinumab and rilonacept.

## Methods

This multi-center observational study included 45 patients (26 children and 19 adults) (study group). At baseline examination, all patients displayed some symptoms suggestive of CAPS. Genetic analysis detected one of the following NLRP3 variants: Q703K (n = 19), R488K (n = 6), and V198M (n = 20).

Clinical presentation was recorded and inflammation markers were analyzed. Data from follow-up visits were also evaluated to assess response to IL-1 inhibitors. Results were compared to a (control) group of CAPS patients (n = 28) in which disease-causing mutations had been confirmed (A439V, E311K, T348M).

## Results

At baseline examination, study patients reported signs of systemic inflammation such as fever (76%), headache (73%), musculoskeletal symptoms (84%) and fatigue (78%). Other CAPS-specific features were rash (80%), conjunctivitis (44%) and sensorineural hearing loss (29%).

Compared to the control group, a history of eye impairment, hearing loss and renal involvement was significantly less frequent in the study group. However study group patients presented significantly more often with gastrointestinal symptoms such as abdominal pain (56% versus 25%, p = 0.01) and gastroesophageal reflux (22% versus 0%, p = 0.01). Also, a wide spectrum of concomitant diseases such as thyroid disorders (7) and neurological and psychiatric diseases were reported (epilepsy (3), Asperger syndrome (2)).

Inflammation markers were only slightly increased: ESR was elevated in 26% (9/35) and C-reactive protein (CRP) in 34% (14/41). Serum amyloid A (SAA) was raised in 36% (8/22) of the patients. Nine out of ten patients (90%) had elevated TNF-α-levels at baseline examination.

Data from follow-up visits during the first year of treatment was available from 20 patients, treated with IL1 - inhibitors. Clinical disease activity was reduced in all cases; 10 patients (50%) achieved full remission and 10 patients showed partial response to the treatment with mild disease activity and/ or persistently elevated inflammation markers.

## Conclusion

Heterozygous carriers of the NLRP3 variants V198M, R488K, and Q703K display distinct clinical characteristics compared to CAPS patients with confirmed disease causing mutations, including a high incidence of gastrointestinal symptoms, only slightly elevated inflammatory parameters, and a potentially inferior response to IL-1 inhibition. Also susceptibility for concomitant diseases is observed.

## Disclosure of interest

None declared.

